# Olfactory Function Recovery in Endoscopic Transsphenoidal Pituitary Surgery

**DOI:** 10.1002/alr.70015

**Published:** 2025-08-11

**Authors:** Sanjena Venkatesh, Jadyn Wilensky, Maria Espinosa, Alison J. Yu, Michael A. Kohanski, James N. Palmer, Nithin D. Adappa, Jennifer E. Douglas

**Affiliations:** ^1^ Perelman School of Medicine at the University of Pennsylvania Philadelphia Pennsylvania USA; ^2^ Department of Otorhinolaryngology – Head and Neck Surgery University of Pennsylvania Philadelphia Pennsylvania USA

**Keywords:** objective olfaction, olfactory outcomes, olfactory quality of life, pituitary adenoma, transsphenoidal approach

## Abstract

- Olfactory flap preservation in transsphenoidal surgery (TSA) promotes early olfactory recovery.
- Objective olfactory function recovers faster than subjective olfaction following TSA.
- Olfactory‐specific and sinonasal olfactory‐specific quality of life (QOL) smell scores show divergent recovery timelines.

## 1 Introduction

Pituitary adenomas are relatively common tumors of the anterior skull base. While typically benign, these masses can compress local structures, leading to headaches, visual deficits, pituitary dysfunction, and more. The endoscopic transsphenoidal approach (TSA) has become standard of care in surgical management of pituitary adenomas, offering enhanced visualization and reduced morbidity over open craniotomy [1]. However, concerns persist regarding the impact of transnasal approaches on olfactory function, particularly with nasoseptal flap (NSF) harvest. Olfactory flap (OF) preservation has emerged as a promising strategy for maintaining postoperative olfactory function. This study aims to elucidate recovery patterns for objective olfactory function, olfactory‐specific quality of life (QOL), and sinonasal QOL following OF‐preserving TSA for pituitary adenoma.

## 2 Methods

### 2.1 Study Design

We conducted a prospective cohort study enrolling all patients undergoing OF‐preserving TSA for pituitary adenoma at our institution between July 2024 and April 2025. The electronic medical record was reviewed for patient demographics. Objective olfaction was assessed using the Brief Smell Identification Test (B‐SIT), a validated 12‐item scratch‐and‐sniff questionnaire [2]. To assess olfactory‐specific and sinonasal QOL, patients were administered the Brief Questionnaire of Olfactory Disorders—Negative Statements (B‐QOD) and the 22‐item SinoNasal Outcome Test (SNOT‐22), respectively. The B‐QOD is a subjective olfaction tool consisting of seven statements that evaluate the degree to which patients suffer from their smell loss [3]. The SNOT‐22, a widely used and validated instrument, measures the impact of sinonasal symptoms on health‐related QOL [4]. Within the SNOT‐22, a single item assesses olfactory function, referred to as the “SNOT smell score” in our analysis. All assessments were conducted pre‐operatively, as well as serially postoperatively to 6 months. Statistical analysis was performed using Prism.

### 2.2 Surgical Technique

All patients underwent transsphenoidal approach to the skull base in the standard fashion. For reconstruction, an NSF was elevated with OF preservation: the traditional flap design was modified to place the superior septal incision 1.5‐2 centimeters below the skull base, as previously published [5].

## 3 Results

Sixty‐five patients (mean age 51.7 years, 41.5% male) underwent OF‐preserving TSA for pituitary adenoma. Baseline characteristics are highlighted in Table 1.

**TABLE 1:** Baseline cohort characteristics.

Characteristic	Pituitary adenoma (n = 65)
Age, mean ± SD	51.7 ± 16.1
Male, *n* (%)	27 (41.5%)
Race, n (%)	
White	38 (58.5%)
Black	16 (24.6%)
Asian	5 (7.7%)
Other/unknown	6 (9.2%)
B‐SIT, mean ± SD	9.6 ± 2.0
B‐QOD, mean ± SD	0.0 ± 0.0
SNOT‐22 smell question, mean ± SD	0.3 ± 0.7
Total SNOT‐22, mean ± SD	18.4 ± 16.0

**FIGURE 1:**
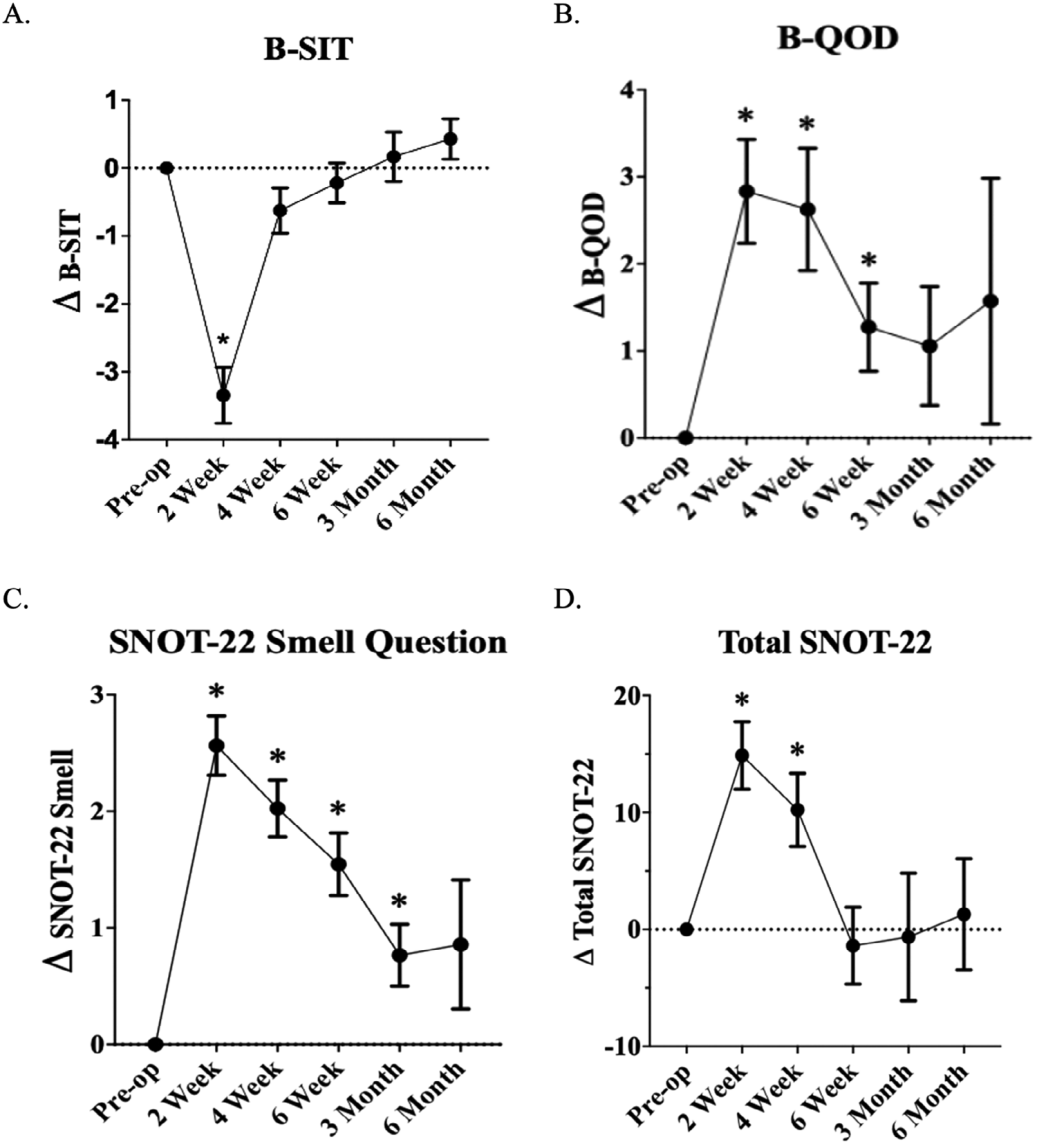
Baseline and serial postoperative metrics. (A) B‐SIT, (B) B‐QOD, (C) SNOT‐22 smell, and (D) SNOT‐22.

Objective olfaction, as measured by B‐SIT scores, demonstrated the earliest recovery. Scores showed significant deficits at 2 weeks (Δ−3.3 ± 3.1, *p* < 0.0001) post‐surgery but returned to clinical and statistical baseline by 4 weeks (Δ−0.6 ± 2.1, *p* = 0.07) (Figure 1A). Of note, the minimum clinically important difference (MCID) in B‐SIT scores is one point [6].

Subjective olfaction measures displayed more prolonged recovery trajectories. B‐QOD scores showed significant impairment at 2 (Δ+2.8 ± 4.4, *p* < 0.0001) and 4 weeks (Δ+2.6 ± 4.4, *p* = 0.0006), returning to clinical baseline at 6 weeks (Δ+1.3 ± 2.9, *p* = 0.02) and statistical baseline at 3 months (Δ+1.1 ± 2.9, *p* = 0.14) (Figure 1B). The MCID for the B‐QOD can be approximated as 2.14, based on its proportion to that of the standard [7]. SNOT smell scores returned to baseline even later at 6 months (Δ+0.9 ± 1.5, *p* = 0.17), with significant deficits at 2 weeks (Δ+2.6 ± 1.9, *p* < 0.0001), 4 weeks (Δ+2.0 ± 1.6, *p* < 0.0001), 6 weeks (Δ+1.5 ± 1.5, *p* < 0.0001), and 3 months (Δ+0.8 ± 1.1, *p* = 0.01) (Figure 1C).

General sinonasal QOL, as measured by the SNOT‐22, showed significant deterioration at 2 (Δ+14.9 ± 21.4, *p* < 0.0001) and 4 weeks (Δ+10.2 ± 20.0, *p* = 0.002), with return to clinical and statistical baseline by 6 weeks (Δ−1.4 ± 18.9, *p* = 0.67) post‐surgery (Figure 1D). The MCID for SNOT‐22 scores is nine points [8].

## 4 Discussion

This study provides a systematic evaluation of objective and subjective olfactory and sinonasal recovery following OF‐preserving TSA. We demonstrate early return to baseline across domains, reinforcing the value of OF preservation in supporting robust recovery.

Our findings align with prior research. Previous studies similarly report return to baseline objective olfaction and sinonasal health by 6 months following OF‐preserving surgery [5, 9]. Our study, however, offers several key strengths that distinguish it from existing literature. First, we focus on an exclusive cohort of pituitary adenoma patients, minimizing confounding seen in prior studies that include more heterogeneous populations (i.e., meningiomas, craniopharyngiomas). Second, we investigate outcomes of the B‐QOD—an olfactory‐specific QOL metric that has not been previously examined in the context of skull base surgery. Finally, we provide an unprecedented level of granularity, with data collection at several postoperative timepoints. This high‐resolution, serial data capture offers unique insights into the trajectory of olfactory recovery.

We find, in our cohort, that objective olfaction recovers significantly faster than subjective olfaction. This observation mirrors findings in other clinical scenarios involving olfactory dysfunction, such as post‐COVID recovery [10]. Together, these results reflect the complex, multidimensional nature of smell and highlight the importance of using both objective and subjective measures when evaluating olfaction.

Regarding subjective olfaction, the notable discrepancy between B‐QOD and SNOT smell score recovery timelines raises important methodological considerations for future research. The B‐QOD is a validated olfactory‐specific QOL instrument that provides a multidimensional assessment of smell. In contrast, the SNOT smell score consists of a single item. The inconsistency between metrics challenges the historical reliance on the SNOT smell score as a proxy for subjective olfactory function, suggesting that this measure may not effectively capture olfactory‐specific QOL.

This study has two key limitations. First, the B‐SIT and B‐QOD have not been specifically validated in the context of endoscopic skull base surgery. Second, this study lacks a comparative group. While our findings suggest favorable olfactory recovery with OF preservation, we cannot definitively attribute these outcomes to the surgical technique itself. Future studies incorporating a comparative design are needed to validate the benefits of OF preservation and more clearly delineate its role in postoperative olfactory recovery.

## Conflicts of Interest

The authors declare no conflict of interest.
